# Metabolic Engineering for Glycyrrhetinic Acid Production in *Saccharomyces cerevisiae*

**DOI:** 10.3389/fbioe.2020.588255

**Published:** 2020-11-19

**Authors:** Ruobing Guan, Mengge Wang, Zhonghua Guan, Cheng-Yun Jin, Wei Lin, Xiao-Jun Ji, Yongjun Wei

**Affiliations:** ^1^State Key Laboratory of Wheat and Maize Crop Science, College of Plant Protection, Henan Agricultural University, Zhengzhou, China; ^2^Key Laboratory of Advanced Drug Preparation Technologies, Ministry of Education, School of Pharmaceutical Sciences, Zhengzhou University, Zhengzhou, China; ^3^School of Basic Medical Sciences (Zhongjing School), Henan University of Chinese Medicine, Zhengzhou, China; ^4^Department of Microbiology and Immunology, School of Medicine & Holistic Integrative Medicine, Nanjing University of Chinese Medicine, Nanjing, China; ^5^College of Biotechnology and Pharmaceutical Engineering, Nanjing Tech University, Nanjing, China

**Keywords:** glycyrrhetinic acid, *Saccharomyces cerevisiae*, metabolic engineering, microbial cell factories, natural product production

## Abstract

Glycyrrhetinic acid (GA) is one of the main bioactive components of licorice, and it is widely used in traditional Chinese medicine due to its hepatoprotective, immunomodulatory, anti-inflammatory and anti-viral functions. Currently, GA is mainly extracted from the roots of cultivated licorice. However, licorice only contains low amounts of GA, and the amount of licorice that can be planted is limited. GA supplies are therefore limited and cannot meet the demands of growing markets. GA has a complex chemical structure, and its chemical synthesis is difficult, therefore, new strategies to produce large amounts of GA are needed. The development of metabolic engineering and emerging synthetic biology provide the opportunity to produce GA using microbial cell factories. In this review, current advances in the metabolic engineering of *Saccharomyces cerevisiae* for GA biosynthesis and various metabolic engineering strategies that can improve GA production are summarized. Furthermore, the advances and challenges of yeast GA production are also discussed. In summary, GA biosynthesis using metabolically engineered *S. cerevisiae* serves as one possible strategy for sustainable GA supply and reasonable use of traditional Chinese medical plants.

## Introduction

Licorice refers to the legume plant of *Glycyrrhiza uralensis* Fisch., *Glycyrrhiza flata* Bat. or *Glycyrrhiza glabra* L., and it is widely used in traditional herbal medicine (Asl and Hosseinzadeh, 2008). Glycyrrhetinic acid (GA) is one of the main bioactive components of licorice plants and it has two optical isomers, 18 α-GA and 18 β-GA (Zhang and Ye, 2009; Li et al., 2014). Between the two isomers, the 18 α-GA isomer is present at low levels. The 18 β-GA is found at higher levels, and it is the main bioactive licorice component. 18 β-GA has a remarkably broad spectrum of biological activities, including as an anti-inflammatory agent (Kowalska and Kalinowska-Lis, 2019), an anti-tumor agent (Wang et al., 2017; Zhou et al., 2019), an immunomodulatory agent (Ayeka et al., 2016), a hepatoprotective agent (Mahmoud and Dera, 2015), and other functional medical effects (Chen et al., 2013; Ding et al., 2020).

Currently, GA is mainly produced by the hydrolysis of glycyrrhizic acid, which is extracted from the dried root and rhizome of licorice (Mukhopadhyay and Panja, 2008; Wang C. et al., 2019). Wild licorice, however, has been severely damaged and has nearly disappeared due to excessive mining (Shu et al., 2011). Moreover, the quality of artificially cultivated licorice is variable, and the GA content in natural licorice is low. The complex structure of GA makes the chemical synthesis of GA difficult (Wang et al., 2018). The demand for GA has increased and the price of GA is high, so exploring other sustainable sources of GA is of great interest.

Introducing the biosynthetic genes of natural products into microbial chassis cells and the construction of microbial cell factories to obtain high levels of targeted natural products have become one of the most essential ways to produce plant natural products (Pyne et al., 2019). The development of synthetic biology and metabolic engineering makes the microbial biosynthesis of GA possible (Wang C. et al., 2019), and microbial cell factories provide a practical and effective way to solve the problem of sustainable production of medical plant resources (Xu et al., 2020).

Microorganisms can utilize a relatively cheap carbon source to balance cell growth and natural product synthesis (Jeandet et al., 2013; Xu et al., 2020). Normally, microorganisms have known genetic background, and there are many ways to genetically manipulate them, making them suitable for use in large-scale fermentation (Xu et al., 2020). At present, there are a variety of natural products, such as artemisinin (Paddon et al., 2013), protopanaxadiol (Dai et al., 2013), ginsenosides (Yan et al., 2014; Wang et al., 2015; Wei et al., 2015), tanshinones (Guo et al., 2016), cocoa butter (Wei et al., 2017a; Bergenholm et al., 2018; Wei et al., 2018), and cannabidiol acid (Luo et al., 2019), that have been successfully synthesized using metabolically engineered *Saccharomyces. cerevisiae*.

In this review, we summarize the current progress for GA production in *S. cerevisiae* and provide some future prospects for the field.

## Chemical Structure of GA

GA is an oleanane-type triterpenoid, and it is soluble in ethanol, methanol, chloroform, ethyl acetate, pyridine and acetic acid, but is insoluble in water (Wang et al., 2018; Kowalska and Kalinowska-Lis, 2019). The chemical structure of GA is complex, hence its chemical synthesis is difficult (Wang C. et al., 2019). The hydroxy group at position C-3 and the carboxyl group at position C-30 are the main functional groups. Currently, chemical strategies are mainly used for modification of GA (Yang et al., 2020). Introduction of protected or de-protected amino acids have been shown to increase GA activity (Schwarz et al., 2014). By esterification of the C-3 and C-30 positions in GA, different GA groups can be introduced and other derivatives can be formed, and these derivatives have been shown to improve the activities of the GA-derived components (Maitraie et al., 2009; Schwarz and Csuk, 2010; Yang et al., 2020). Nowadays, the cost of chemical synthesis of GA is high, while the yield is low (Wang C. et al., 2019). Additionally, large amounts of wastes are generated during chemical synthesis of GA.

## GA Biosynthetic Pathway

The production of a plant natural product in microorganisms requires an understanding of its biosynthetic pathways (Wei et al., 2019). Traditional pathway mining strategies are typically carried out through selection of mutant libraries, which is time-consuming and laborious. With the development of bioinformatics and computational biology, it has been possible to identify all potential biosynthetic pathways of the natural products in one species by establishing a multi-layered omics technology platform (Mochida and Shinozaki, 2011). The platform includes omics data analyses of transcripts, genomes, proteomes, metabolomes, the prediction of gene function, and the verification of key genes in the biosynthetic pathways of natural products (Nett et al., 2020).

**FIGURE 1:**
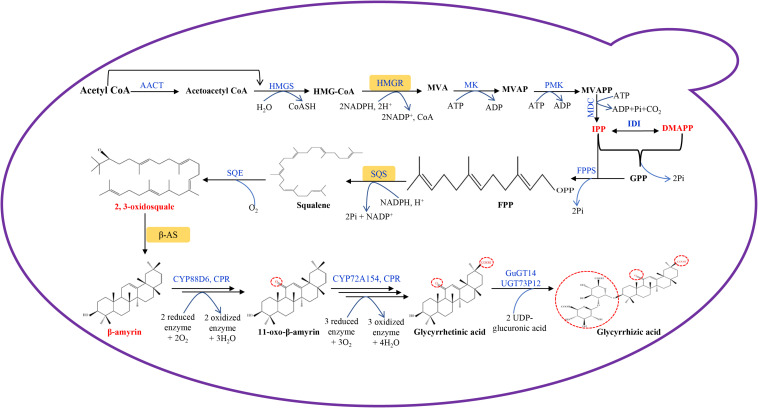
Glycyrrhetinic acid biosynthetic pathway designed in engineered *S. cerevisiae*. AACT, acetyl-CoA C-acetyltransferase; HMGS, 3-hydroxy-3- methylglutaryl-CoA synthase; HMGR, 3-hydroxy-3-methylglutaryl-CoA reductase; MK, mevalonate kinase; PMK, phosphomevalonate kinase; MDC, mevalonate diphosphate decarboxylase; GPP, geranyl diphosphate; GPPS, geranyl diphosphate synthase; IDI, isopentenyl-pyrophosphate delta isomerase; FPP, farnesyl diphosphate; FPPS, farnesyl diphosphate synthase; SQS, squalene synthase; SQE, squalene epoxidase; β-AS, β-amyrin synthase; CPR, cytochrome P450 reductase; GuGUAT, *Glycyrrhiza uralensis* UDP-dependent glucuronosyltransferases; MVA, mevalonic acid; MVAP, mevalonate phosphate; MVAPP, mevalonate diphosphate; IPP, isopentenyl pyrophosphate; DMAPP, dimethylallyl pyrophosphate.

Transcriptomic and genomic sequencing data are available for licorice (Li et al., 2010; Ramilowski et al., 2013; Mochida et al., 2017; Gao et al., 2020), and have contributed to a better understanding of natural product biosynthetic pathways in licorice plants. The GA biosynthetic pathway in licorice plants has been defined (Seki et al., 2011). GA belongs to the oleanane-type triterpenoid group, and shares the same precursor synthetic pathway with other terpenes. Both methylerythritol phosphate (MEP) and mevalonate (MVA) pathways are involved in GA precursor synthesis, but GA is mainly synthesized through the MVA pathway which can be divided into three stages (Figure 1).

In the first stage, acetyl-CoA is catalyzed by acetyl-CoA C-acetyltransferase (AACT), 3-hydroxy-3-methylglutaryl-CoA synthase (HMGS), and 3-hydroxy-3-methylglutaryl-CoA reductase (HMGR) to generate MVA, followed by multi-step reactions to synthesize isopentenyl pyrophosphate (IPP) and its isomer dimethylallyl pyrophosphate (DMAPP). HMGR is the first key rate-limiting enzyme in the MVA metabolic pathway, in which HMGR can catalyze the formation of MVA from HMG-CoA, which is an irreversible reaction (Friesen and Rodwell, 2004). IPP and DMAPP have been shown to be the important intermediate products of this process, and they are the precursors for terpenoids synthesis in plant cells (Wang Q. et al., 2019).

Secondly, following the catalysis of geranyl diphosphate synthase (GPPS), farnesyl diphosphate synthase (FPPS), squalene synthase (SQS) and squalene epoxidase (SQE), IPP is used to synthesize 2,3-oxidosquale. 2,3-oxidosquale is a common precursor for some natural product biosynthesis, such as sterols and triterpenes. SQS synthesize squalene using farnesyl diphosphate (FPP) as substrate, and it has been identified to be a key enzyme which can direct carbon flow to triterpene biosynthesis (Seo et al., 2005).

2,3-oxidosquale is catalyzed by β-amyrin synthease (bAS) to generate β-amyrin, which is an important reaction step in GA biosynthesis. Following this, the licorice enzyme CYP88D6 catalyzes the hydroxylation of the 11-position of β-amyrin, followed by further oxidation to 11-oxo-β-amyrin (Seki et al., 2008). Licorice CYP72A154 further catalyzes a three-step reaction. The C-30 position of 11-oxo-β-amyrin is firstly hydroxylated, then the product has been shown to be oxidized to form an aldehyde, and finally it is again oxidized to form the carboxyl group of GA (Seki et al., 2011). During this process, NADPH-cytochrome P450 reductase (CPR) is the main electron donor to cytochrome P450 (CYP450). The electrons are transferred to CYP450, followed by a redox reaction with the substrate (Zhu et al., 2018). The electron transfer reaction between CPR and CYP450 is the rate-limiting step of the CYP450 redox reaction, which affects GA synthetic efficiency (Zhu et al., 2018). GA is converted to glycyrrhizic acid by the UDP-dependent glucuronosyltransferases of GuGT14 and UGT73P12 (Chen et al., 2019; Nomura et al., 2019).

## Metabolic Engineering of *S. cerevisiae* for GA Production

*S. cerevisiae* is one of the most commonly used model chassis, in particular grows rapidly on low-cost substrates and genetic manipulation is easy. Besides, the MVA pathway in *S. cerevisiae* can provide precursors like IPP and DMAPP for terpene synthesis. Many natural products have been successfully synthesized in *S. cerevisiae*. After a series genetic modification to enhance the MVA pathway in *S. cerevisiae*, the production of artemisinin can reach 300 mg/L. Further optimization of the metabolic network and fermentation conditions increased the titer to 25 g/L (Paddon et al., 2013). The annual artemisinin output in a 100 m^3^ fermentation bioreactor is 35 tons, which is equivalent to the output of artemisinin of 50,000 mu in *Artemisia annua*, showing that yeast cell factories can provide large amounts of natural products in a short time and with limited space.

By introducing more than 20 genes from plants, bacteria or rodents into *S. cerevisiae*, an artificial *S. cerevisiae* cell factory that can convert sugars into opioid alkaloids has been successfully constructed, representing a breakthrough in the biosynthesis and manufacturing of opioid alkaloids (Galanie et al., 2015). Additionally, the complete microbial synthesis of major cannabinoids and their derivatives, including cannabigerol acid (CBGA), tetrahydrocannabinolic acid (THCA) and cannabidiol acid (CBDA), in *S. cerevisiae* cell factories has been achieved (Luo et al., 2019). The synthesis of GA requires the MVA pathway to provides precursor substances. *S. cerevisiae* can be used as an advantageous chassis cell to supply substrates for GA synthesis, and various metabolic engineering strategies had been used for GA production in *S. cerevisiae*.

β-amyrin serves as the oleanane-type triterpenoid precursor to various downstream products, and increasing β-amyrin synthesis in *S. cerevisiae* is essential for GA synthesis. The *AaBAS* gene cloned from *A*. *annua* was introduced into *S. cerevisiae*, which altered the gene expression level of HMGR and lanosterol synthase gene (ERG7), increasing β-amyrin production in *S. cerevisiae* by 50% to 6 mg/L (Supplementary Table 1; Kirby et al., 2008). This result shows that triterpenes can be produced in *S. cerevisiae*, and a heterologous gene from another plant could increase β-amyrin production. Subsequently, Dai et al. (2015) integrated two different *bAS* genes derived from *Glycyrrhiza glabra* and *Panax ginseng*, and another copy of native yeast SQS and SQE genes in yeast. Following incubation in YPD medium with 2% glucose for 7 days, Gas chromatography–mass spectrometry (GC/MS) analysis showed that the β-amyrin production yield was 107 mg/L with a yield of 9.3 mg/g dry cell weight (DCW) (Supplementary Table 1; Dai et al., 2015). These studies indicated that overexpression of key genes in the GA biosynthetic pathway could increase precursor production.

A combination of stepwise rational refactoring and directed flux regulation strategies can improve β-amyrin production. Introduction of the *bAS* gene from *Glycyrrhiza glabra* and the heterologous squalene monooxygenase genes from *Candida albicans* into *S. cerevisiae*, combined with overexpression of isopentenyl pyrophosphate isomerase (IPI), FPPS and SQS genes, increased squalene production. UPC2 is a global transcription factor regulating sterol synthesis in *S. cerevisiae*, and modifying the UPC2 binding site directed metabolic flux for β-amyrin biosynthesis. After a series of genetic modifications and suitable ethanol fed-batch fermentation, the final titer of β-amyrin in *S. cerevisiae* reached 138.80 mg/L (Zhang et al., 2015). This suggested that pathway optimization and flux refactoring can improve natural product synthesis in engineered yeast. Liu et al. introduced an optimal acetyl-CoA pathway and deleted an acetyl-CoA competing pathway in *S. cerevisiae*, balancing various factors that greatly reduced energy consumption and glucose utilization. The final β-amyrin production was found to be 279.0 ± 13.0 mg/L, which is the highest β-amyrin production ever reported (Supplementary Table 1; Liu et al., 2019). This study suggested that a global analysis between precursor supply and product formation, rather than analysis of one key gene, could efficiently improve natural product biosynthesis in engineered microorganisms. In the GA biosynthesis pathway, β-amyrin is further catalyzed by CYP88D6 and CYP72A154 to synthesize GA (Supplementary Table 1; Seki et al., 2008, 2011).

Seki et al. (2011) also confirmed the key roles played by CYP450 genes in the GA biosynthesis pathway by introducing bAS, CYP88DE, CYP72A154, and CPR into wild-type *S. cerevisiae*, thus redirecting the metabolic flow to GA synthesis. This eventually led to 11-oxo-β-amyrin production levels of 76 μg/L and GA production amounts of 15 μg/L (Supplementary Table 1; Seki et al., 2011). These results indicated the feasibility of GA synthesis in yeast using synthetic biology. It is possible to improve GA production by enhancing the catalytic step in which CYP72A154 converts 11-oxo-β-amyrin to GA. Zhu et al. further balanced oxidation and reduction systems by introducing two novel CYP450 genes, Uni25647 and CYP72A63, and pairing the new cytochrome P450 reductases GuCPR1 from *Glycyrrhiza uralensis*. With the optimized oxidation and reduction modules, as well as scale-up fed-batch fermentation, 11-oxo-β-amyrin and GA synthesis reached 108.1 ± 4.6 mg/L and 18.9 ± 2.0 mg/L, respectively (Zhu et al., 2018). These are the highest titers ever reported for GA and 11-oxo-β-amyrin synthesized from *S. cerevisiae*. This work further showed that redox balance mediated by the CYP450 and CPR genes was important for GA synthesis. Wang et al. directed the upstream carbon flux flow from acetyl-CoA to 2,3-oxidosqualene by introducing a newly discovered gene, cytochrome b5, from *Glycyrrhiza uralensis* (GuCYB5) and overexpressing 10 known MVA pathway genes (*ERG20*, *ERG9*, *ERG1*, *tHMG1*, *ERG10*, *ERG8*, *ERG13*, *ERG12*, *ERG19*, *IDI1*) of *S. cerevisiae*. The shaking flask titer of 11-oxo-β-amyrin and GA increased to 80 and 8.78 mg/L, respectively (Supplementary Table 1; Wang C. et al., 2019).

**FIGURE 2:**
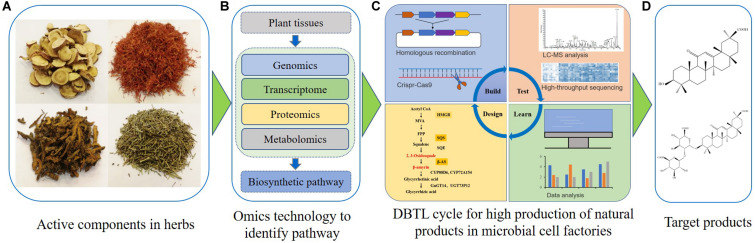
Engineering microbial cell factories for synthesis of glycyrrhetinic acid or other plant natural products. **(A)** The active components are identified in traditional Chinese herbs or other candidate plants. **(B)** The fresh samples are collected, and multi-omics technologies are used to discover the biosynthetic pathways of natural products. **(C)** The Design-Build-Test-Learn cycle is used for construction and optimization of microbial cell factories for natural product biosynthesis. **(D)** Separation and purification of targeted natural products.

Numerous studies have recently shown the feasibility of GA synthesis in *S. cerevisiae* (Zhu et al., 2018; Wang C. et al., 2019). According to published results, the microbial synthesis of GA and other natural products might be achieved by several key procedures (Figure 2). First, plants harboring GA or other plant bioactive components are identified. Secondly, transcriptomics, genomics, proteomics and metabolomics analyses are used to recover pathways and key enzymes related to the synthesis of GA or other natural products (Chen et al., 2020; Gao et al., 2020; Nett et al., 2020; Srinivasan and Smolke, 2020). After that, appropriate chassis cells which provide precursors for natural product synthesis are selected for construction and optimization of associated microbial cell factories. Some synthetic biology tools, such as CRISPR-Cas9 technology, can efficiently introduce the biosynthetic pathway for GA or other natural products into chassis cells. These chassis cells may then be used as microbial cell factories. The artificially constructed cell factories need to be continuously optimized through the Design-Build-Test-Learn (DBTL) cycle (Nielsen and Keasling, 2016). The DBTL cycle includes new enzyme discovery, heterologous gene expression, promoter engineering, metabolic flux balance, pathway optimization, oxidation and reduction system balance, genome-scale metabolic models and other metabolic engineering strategies (Jiang et al., 2020; Ko et al., 2020; Tang et al., 2020; Wang M. et al., 2020). Finally, a high-yield GA or other natural product microbial cell factory can be obtained. As GA is insoluble in water, strategies utilizing the harnessing of lipid metabolism or other sub-organelle metabolism could lead to a high-yield production of GA (Cao et al., 2020; Ma et al., 2019). Besides *S. cerevisiae*, other yeasts could also be optimized for natural product biosynthesis in the future (Wei et al., 2017b; Wang J. et al., 2020).

## Conclusion and Future Prospectives

GA is widely used in clinical practices, but the supply of GA is limited through traditional planting methods. Metabolic engineering and synthetic biology tools provide possible strategies for efficiently producing GA in *S. cerevisiae*. The successful production of high β-amyrin and GA using engineered *S. cerevisiae* shows that yeasts are suitable microbial chasses for GA production. The DBTL and other engineering strategies would further increase GA production, possibly providing one sustainable GA supply. In the future, other natural products derived from traditional Chinese herbs or other plants could also be synthesized using engineered *S. cerevisiae*.

## Author Contributions

YW and RG conceived this study. YW, RG, MW, and ZG wrote the manuscript. WL, C-YJ, and X-JJ revised the manuscript. All authors contributed to the article and approved the submitted version.

## Conflict of Interest

The authors declare that the research was conducted in the absence of any commercial or financial relationships that could be construed as a potential conflict of interest.
