# Supplementary material for: Metabolic Engineering for Glycyrrhetinic Acid Production in Saccharomyces cerevisiae
Source: Front Bioeng Biotechnol. 2020 Nov 19;8:588255. doi: 10.3389/fbioe.2020.588255 (PMC7710550; doi:10.3389/fbioe.2020.588255)
Supplement: Supplementary file 1 [file Table_1.docx]

Supplementary Table 1: Summary of metabolic engineering for GA production in *S. cerevisiae*.

| Product | Substrate | | Scale | Parental strain | Genetic modification | Titer (mg/L) | Yield (mg/g) | Productivity (mg/L/h) | References | |
| --- | --- | --- | --- | --- | --- | --- | --- | --- | --- | --- |
| β-amyrin | | SC medium | shake flask | *S. cerevisiae* BY4742 | Introduced *AaBAS* gene into *S. cerevisiae* and changed the gene expression level of *HMGR* and *ERG7*. | 6 |  |  | | Kirby et al., 2008 |
| β-amyrin | | YPD medium | shake flask | *S. cerevisiae* BY4742 | Two different β-amyrin synthase genes of *Glycyrrhiza glabra* and *P. ginseng*, with the *S. cerevisiae* squalene synthase and squalene epoxidase genes, were integrated into the of chromosome of strain BY-T1. | 107 | 9.3 | 1.27 | | Dai et al., 2014 |
| β-amyrin | | YPD medium | fed-batch fermentation in a 5 L bioreactor (Minifors, Switzerland) | *S. cerevisiae* INVSc1 | Introduction of bAS gene from *Glycyrrhiza glabra* and heterologous squalene monooxygenase genes from *Candida albicans* into *S.cerevisiae*, combining overexpression of isopentenyl pyrophosphate isomerase (IPI), FPPS and SQS genes increased the squalene production, and modifying the UPC2 binding site directed metabolic flux for β-amyrin biosynthesis. | 138.8 | 16.3 | 0.96 | | Zhang et al., 2015 |
| β-amyrin | | YPD medium | fed-batch fermentation in a 5 L bioreactor (Minifors, Switzerland) | *S. cerevisiae* SGib | Introduced an optimal acetyl-CoA pathway and deleted an acetyl-CoA competing pathway in *S. cerevisiae*, balancing various factors that greatly reduce energy consumption and glucose utilization. | 279.0 ± 13.0 | 4.65±0.22 | 3.32 ± 0.15 mg | | Liu et al., 2019 |
| Glycyrrhetinic acid | | SC-W-L medium | shake flask | *S. cerevisiae* BJ2168 | Introduced bAS, CYP88DE, CYP72A154, and CPR into wild-type *S. cerevisiae.* | 0.015 |  |  | | Seki et al., 2011 |
| Glycyrrhetinic acid | | YPD medium | fed-batch fermentation in a 7.5 L fermentor | *S. cerevisiae* cen.pk2-1D | Introduced two novel CYP450 genes of Uni25647 and CYP72A63 and pairing the new cytochrome P450 reductases GuCPR1 from Glycyrrhiza uralensis. | 8.78 |  |  | | Wang et al., 2019 |
| Glycyrrhetinic acid | | YPD medium | fed-batch fermentation in a 5 L bioreactor | *S. cerevisiae* SGib | Introduced a newly discovered gene cytochrome b5 from Glycyrrhiza uralensis (GuCYB5) and overexpressing 10 known MVA pathway genes of S. cerevisiae. | 18.9 ± 2.0 | 1.6 ± 0.2 |  | | Zhu et al., 2018 |
